# Mental Hospitals and Recruiting: Replacing Attendants by Women Nurses

**Published:** 1915-07-31

**Authors:** 


### MENTAL HOSPITALS AND RECRUITING.

### Replacing Attendants by Women Nurses.

By A MENTAL HOSPITAL MEDICAL OFFICER.

A point of considerable national interest at the present time is raised in the letter from Dr. G. M. Robertson, physician superintendent of the Royal Edinburgh Asylum, which is printed in another column. It raises a question which may entail a drastic change in the administrative work of mental hospitals. Dr. Robertson, while admitting, as is only just, that the response to recruiting demands has already been admirable among attendants in mental hospitals, suggests that there are still many young men who might enlist if substitutes could be found to carry on their work of caring for the insane.

There are many persons of some experience who will, no doubt, express their scepticism in regard to the alternative suggested—" the substitution of female nurses "—yet it should be remembered that when it was proposed to remove the chains which bound the insane, there was a chorus of disapprobation. The belief that the majority of the insane are potentially or actually dangerous is very widespread, and even among those who have charge of mental hospitals it is apparently not extinct. Like many other beliefs, it is not, however, based upon experience. In many instances, at least where trouble of this kind has been evident, the noisiness, violence, and recalcitrancy have resulted from the methods employed in dealing with the patients. Even among the insane it is found that the soft answer turneth away wrath; and peaceful persuasion is more efficient and more humane than coercion.

### Scotland's Experience.

Fortunately the day is past when the procedure of employing female nurses to care for male patients in mental hospitals could be described as an innovation. For fifteen years it has been tried " on a large scale " in Scotland. The untoward results which were prophesied have not justified the prophets of evil: the gloomy prognostications have not proved to be true. But even if there had been drawbacks or mishaps they would not necessarily have sufficed to bring condemnation unless it could have been proved that under the earlier procedure the care and treatment of the insane was generally more successful and more pleasant. No one can truthfully assert this. The feminine influence is even of greater value in the mental than in the general hospital; yet, whilst there is general agreement that the nurse is desirable for the care of male patients in general hospitals, even in official circles there is hesitation in adopting a similar system in mental hospitals, at least in England. In Scotland such progress has been made that Dr. Robertson is able to write that this method " has been all but universally adopted."

It is not, of course, asserted that the services of male attendants can be entirely dispensed with: as far as can be seen at present it will always be necessary to make use of their services to a considerable extent. What is affirmed, however, is that radical alterations can be made in this direction; and if further demands are made upon the male staffs by the exigencies of war this change will be necessitated.

### Registration and Mental Hospitals.

In many mental hospitals the strain is already so great that it has been suggested that no further depletion should be permitted, and that National Registration should not be made applicable to these institutions. Whether such exemption will be allowed or not makes little difference to the question now discussed. If further calls are made upon the employees of mental institutions they may still be replaced by men over military age or incapacitated by physical defect from serving. It is probable, however, that medical superintendents who choose this issue out of their difficulties may not find it altogether satisfactory. If so, they may decide to accept the alternative of extending the confines of the work allotted to nurses. In the latter case there can be little doubt that they will seldom have occasion to regret the step which they have had to take.

So far, moreover, as the patients are concerned, the present writer—who has had the opportunity of working in one of the very few mental hospitals in England where the bulk of the nursing is done by women and where, indeed, three-fourths of the total number of the patients are so nursed—is able to say from experience that they will welcome the change and that the amenities of their institutional life will be enhanced. Thus out of the evil of the war good may come to a singularly afflicted portion of the community. It was during the storms of the French Revolution that Pinel instituted his reforms in the treatment of the insane!

